# Significant Association Between Increased Abundance of Selected Bacterial Lipopolysaccharides and Norovirus Diarrhea Among South African Infants

**DOI:** 10.3390/v17020278

**Published:** 2025-02-17

**Authors:** Lerato P. Kgosana, Mapaseka L. Seheri, Cliff A. Magwira

**Affiliations:** Diarrheal Pathogens Research Unit (DPRU), Department of Medical Virology, Sefako Makgatho Health Sciences University, Pretoria 0208, South Africa; lerato.kgosana@smu.ac.za (L.P.K.); mapaseka.seheri@smu.ac.za (M.L.S.)

**Keywords:** bacterial lipopolysaccharide, TLR4, norovirus diarrhea, abundance, gene expression

## Abstract

Bacterial lipopolysaccharides (LPS) have been shown to promote enteric viral infections. This study assessed whether possessing elevated levels of LPS was associated with norovirus infection. Fecal samples from diarrheic norovirus-positive (DNP) (n = 26), non-diarrheal norovirus-negative (NDNN) (n = 26), asymptomatic norovirus-positive (ANP) (n = 15), and diarrheic norovirus-negative (DNN) (n =15) infants were assayed for selected bacterial LPS by quantitative PCR. The mean levels of selected LPS gene targets were significantly high in DNP infants (6.17 ± 2.14 CFU/g) versus NDNN infants (4.13 ± 2.25 CFU/g), *p* = 0.003. So too was the abundance between DNP and DNN infants (*p* = 0.0023). The levels of selected LPS gene targets were high regardless of whether the infection was symptomatic or asymptomatic, *p* = 0.3808. The average expression of genes coding for selected LPS and their signalling molecule, Toll-like receptor 4 (TLR4), increased 7- and 2.5-fold, respectively, in DNP versus NDNN children. Infants possessing elevated levels of selected LPS-rich bacteria were 1.51 times more likely to develop norovirus diarrhea (95% CI: 1.14–2.01, *p* = 0.004). In conclusion, norovirus infection was associated with abundance of selected bacterial LPS, suggesting a possible role of bacterial LPS in norovirus infection.

## 1. Introduction

Norovirus is one of the leading causes of acute viral gastroenteritis (AGE) worldwide, accounting for over 200,000 deaths and 685 million infections in children under the age of 5 [1]. Several host innate factors have been associated with an individual’s susceptibility to norovirus infection [2]. Recent studies have also indicated the involvement of commensal bacteria in successful norovirus infections. For instance, norovirus was unable to infect mice treated with antibiotics compared to their untreated counterpart [3]. Several hypotheses that explain how gut bacteria influence enteric viral infection exist, including the effects of bacterial lipopolysaccharides [4]. Lipopolysaccharides (LPS), expressed by mostly Gram-negative bacteria, have been shown to promote enteric viral infections [4]. Binding of the viral particle to bacterial LPS increases its thermostability and resistance to inactivation from chemicals [4]. This subsequently enhances the chance for the virus to infect the target cells, possibly by affording the virus time to reach its target cells. Bacterial LPS also facilitate the attachment of the viral particles to the target cells as the binding of enteric viruses, such as poliovirus to specific receptors, was reported to improve in the presence of bacterial LPS, suggesting that the LPS is directly involved in the attachment of enteric viruses to target cells [4].

The viral binding is not limited to bacterial LPS as studies have shown that the viruses also bind to other bacterial components including N-acetylglucosamine-containing surface polysaccharides that are longer than six monosaccharides [5]. N-acelylglucosamine is a main component of bacterial cell wall peptidoglycan that is made up of a sugar backbone consisting of alternating residues of β-(1,4) linked N-acetylglucosamine and N-acetylmuramic acid [6]. When combined with uridine diphosphate (UDP), N-acetylglucosamine is also a precursor for the synthesis of the lipid A of LPS, which is a major part of the outer membrane of Gram-negative bacteria [7].

Bacterial LPS is a tripartite structure consisting of the lipid A that docks the LPS to the outer membrane; core oligosaccharide, which maintain the integrity of the outer membrane; and O antigen polysaccharide or O antigen, which is connected to the core and made up of a polymer of repetitive oligosaccharide units exposed to and interacting with the external environment [8]. While the genes that code for common polysaccharide are conserved, there is little homology in different set of genes responsible for the biosynthesis of the O-specific antigen in different bacteria, making it impossible to target a single gene to quantify the O antigen of the LPS.

Several intestinal Gram-negative bacteria such as *Serratia marcescens*, *Klebsiella pneumoniae*, *Pseudomonas aeruguinosa*, and others are reported to be rich in LPS and other N acetylglucosamine-containing surface polysaccharides [9,10]. However, despite their significance, little is known about the role of the abundance of LPS-rich bacteria and other N acetylglucosamine-containing surface polysaccharides in pediatric norovirus infection. Here, we report that harbouring increased levels of LPS-rich bacteria and other N acetylglucosamine-containing surface polysaccharides can be a risk factor for pediatric norovirus diarrhea.

## 2. Methods

### 2.1. Study Design, Population, and Sampling

This is a case–control study that used stool samples collected from infants attending Oukasie Healthcare Clinic, north of Pretoria for a routine vaccination programme and diarrhea between 2018 and 2020. Diarrhea was defined as having episodes of at least three loose, watery stools within 24 h, along with at least one of the following symptoms: nausea, vomiting, abdominal pain, or high fever [11]. Stool samples were collected from infants of similar age (3.5 and 9 months) after written and informed consent from the parents. The study cases were limited to these specific months as the study controls were enrolled at a vaccination clinic for rotavirus immunization at 3.5 and 9.0 months of age. Study participants were divided into four groups: diarrheic norovirus-positive (DNP), asymptomatic norovirus-positive (ANP), non-diarrheic norovirus-negative (NDNN), and diarrheic norovirus-negative infants (DNN). Infants on antibiotic therapy or treated with antibiotics 4 weeks prior to recruitment were excluded from the study. Ethical clearance was approved by the Sefako Makgatho Health Sciences University Research and Ethics Committee, SMUREC/M199/2020: PG.

Stool samples were collected from the infants’ diapers into sterile plastic bottles. Watery stool samples were collected by squeezing the soiled part of the diaper into the bottle and immediately freezing at −20 °C and transporting to the laboratory within 30 min in cooler boxes packed with ice blocks.

### 2.2. Stool Viral RNA and Bacterial Genomic DNA Extraction

Viral RNA was extracted from 10% (*w*/*v*) stool suspension using a Viral RNA Mini Extraction Kit (Qiagen, Hilden, Germany) following the manufacturer’s instructions with minor modifications [12]. Viral RNA was eluted in 50 µL buffer and stored at −20 °C for short-term storage and −80 °C for long-term storage. DNA was extracted from stool samples using QIAamp Fast DNA Stool Mini Kit (Qiagen, Hilden, Germany) following the manufacturer’s guidelines. A total of 150 µL of fecal DNA was eluted and stored at −80 °C.

### 2.3. Detection of Norovirus in Stool Samples

Stool viral RNA was assayed for the presence of norovirus by reverse transcriptase polymerase chain reaction (RT-qPCR) using Allplex GI-Virus Assay kit (Seegene, Seoul, Republic of Korea) as recommended by the manufacturer. RT-qPCR assay was performed in a Bio-Rad CFX96 Real-Time System (Bio-Rad Laboratories, Hercules, CA, USA) using a 96-well plate (Nunc, Roskilde, Denmark) and the results were analyzed in a Seegene Viewer (Seegene, Seoul, Republic of Korea). A cycle threshold (Ct) value of 40 or below was considered as positive for norovirus [13]. 

### 2.4. Quantitative PCR Assay for Detection of Selected LPS-Rich Bacteria and Sequencing

Bacterial N-acetylglucosamine and selected LPS-rich bacteria were assayed in the stool DNA samples by qPCR using bacterial N-acetylglucosamine and LPS species-specific primers and probes (this study) (Table 1) that targeted the genes coding for synthesis of bacterial N-acetylglucosamine (GlmU) and LPS of *K. pneumonia* (*Waa*E), *S. marcescens* (*kdt*X), and *P. aeruginosa* (*Waa*L).

Quantitative PCR amplification was performed in a Bio-Rad CFX96 Real-Time System (Bio-Rad Laboratories, Hercules, CA, USA) using 8-tube PCR strips (Bio-Rad Laboratories, Watford, UK). The 20 µL reaction mix was made up of 2X Luna Universal qPCR Master Mix (New England BioLabs, Ipswich, MA, USA), 0.25 µM each of the probe, forward and reverse primer, 5 µL bacterial DNA, and nucleic free water. The qPCR assay was performed under the following conditions: initial denaturation at 95 °C for 5 min, 45 cycles of 95 °C for 15 s, and 60 °C for 30 s. Conventional PCR products obtained by amplification of selected gene targets using species specific primers (Table 1) were sanger-sequenced (Inqaba Biotech, Pretoria, South Africa) to confirm their identity.

### 2.5. Bacterial Quantification Standards

The standard curves used to quantify the selected LPS-rich bacteria were prepared from genomic DNA extracted from *K. pneumoniae* (ATCC: BAA-2146), *S. marcescens* (ATCC: 43862), and *P. aeruginosa* (ATCC: 27853). In summary, for each bacterial strain used, cultures were inoculated in Luria–Bertani broth (Gibco, Dublin, Ireland) until the strains were in the logarithmic growth phase. Tenfold serial dilutions were prepared and plated in triplicate on nutrient agar plates to enumerate the bacterial colony-forming units (CFU). The remainder of each serial dilution was used to extract the DNA. The extracted DNA was used to generate standard curves by plotting the qPCR Ct value of each dilution against the corresponding CFU.

### 2.6. Bacterial mRNA Extraction and cDNA Synthesis

Bacterial mRNA was extracted from 10% stool (*w*/*v*) suspension using NucleoSpin Mini RNA kit (Machinery Nagel, Duran, Germany) following the manufacturer’s instructions. Complementary DNA (cDNA) was synthesized using Tetro cDNA Synthesis kit (Bioline Inc., Tauton, MA, USA). A 20 µL reaction mix consisted of 1 µL random hexamers, 1 µL of 10 mM dNTPs mix, 4 µL of 5x RT Buffer, 1 µL RiboSafe RNase Inhibitor, 200 u of reverse transcriptase, 7 µL RNase-free water, and 5 µL RNA template. The reverse transcriptase reaction was performed under the following conditions: 25 °C for 10 min, followed by 45 °C for 30 min, and 85 °C for 5 min; the reaction was immediately chilled on ice and stored at −20 °C until further use.

### 2.7. LPS and TLR4 Gene Expression

The expression of the selected LPS and TLR4 genes in stool mRNA samples was determined by real-time PCR using HOT FIREPol^®^ EvaGreen^®^ qPCR Mix Plus (ROX) (Solis BioDyne, Tartu, Estonia) and primers listed in Table 1 as recommended by the manufacturer with minor modifications. Briefly, the 20 µL reaction mixture consisted of 1X HOT FIREPol^®^ EvaGreen^®^qPCR Mix Plus, 400 nM of both forward and reverse primers, 5 µL of the cDNA template and the remainder was made PCR grade water. The qPCR was performed in Bio-Rad CFX96 Real-Time System (Bio-Rad Laboratories, Hercules, CA, USA) under the following conditions: 95 °C for 5 min and 45 cycles of 95 °C for 10 s and 60 °C for 15 s. A melting curve was included in the analysis to differentiate between true positives and primer dimers. Values of the LPS and TLR4 gene expressions were normalized against the housekeeping GAPDH and calculated as previously described [14]. The difference in gene expression was presented as fold change.

### 2.8. Statistical Analysis

Differences in demographics between the diarrhea norovirus-positive and non-diarrhea norovirus-negative infants were determined by the χ^2^ test. The bacterial CFU/g counts were converted to a logarithmic form while descriptive statistics were presented in terms of mean and standard deviation and displayed as box plots using Graphpad Prism 9.2.0 (GraphPad Software, San Diego, CA, USA). A Mann–Whitney U test was used to determine differences in levels of selected LPS-rich bacteria between the study groups. Bacterial CFU/g were used as a continuous variable to test the association between bacterial levels of selected LPS-rich bacteria and norovirus diarrhea using logistical regression. Multiple logistical regression analysis was also used to assess the contribution of potential confounding factors in predicting levels of the selected LPS-rich bacteria and bacteria N-acetylglucosamine. The difference in fold expression of the selected genes was presented as mean and standard deviation and was analyzed with a parametric unpaired *t*-test with Welch’s correction. The normality in the distribution of the data was determined with the Kolmogorov–Smirnov test. A *p*-value less than or equal to 0.05 was considered significant.

## 3. Results

### 3.1. Demographics and Other Characteristics

No significant differences were observed in demographics and other characteristics between diarrheic norovirus-positive and non-diarrheic norovirus-negative groups (Table 2). Similarly, there were no significant differences in the demographics between asymptomatic norovirus-positive and diarrheic norovirus-negative infants.

### 3.2. Abundance of Selected LPS-Rich Bacteria and N-Acetylglucosamine in Diarrheic Norovirus-Positive and Non-Diarrheic Norovirus-Negative Infants

**Figure 1:**
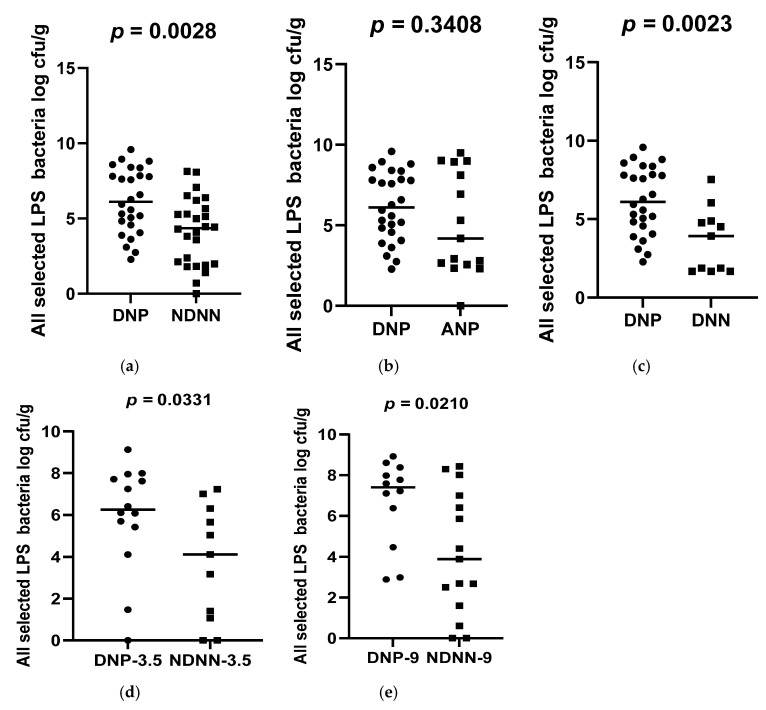
Average abundance of all selected LPS-rich bacteria in (**a**) DNP vs. NDNN (**b**) DNP vs. ANP (**c**) DNP vs. DNN (**d**) DNP vs. NDNN at 3.5 months of age, (**e**) DNP vs. NDNN at 9 months of age. — denotes the mean.

**Figure 2:**
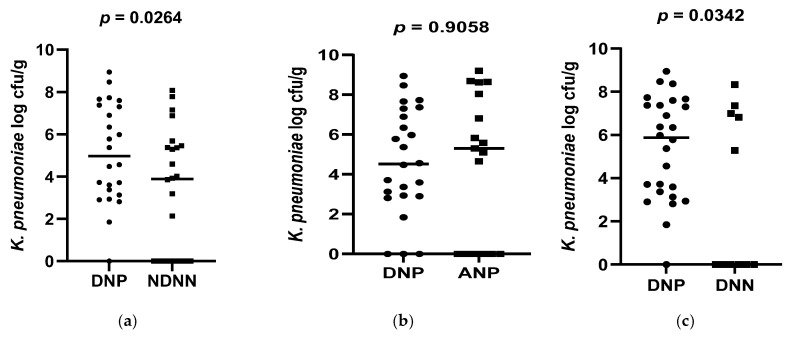

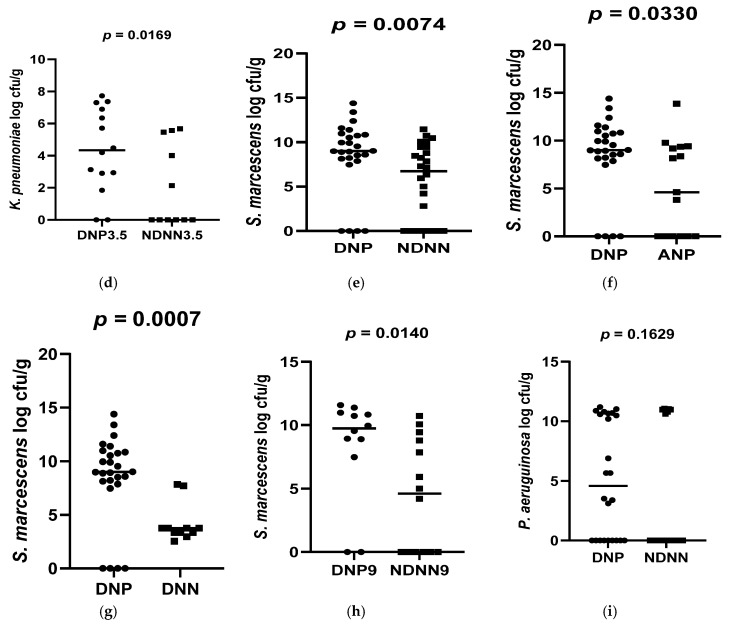
Abundance of each of the selected LPS-rich bacteria in (**a**) DNP vs. NDNN (*K. pneumonia*), (**b**) DNP vs. ANP (*K. pneumonia*), (**c**) DNP vs. DNN (*K. pneumonia*), (**d**) DNP vs. NDNN (*K. pneumonia*) at 3.5 months old (**e**) DNP vs. NDNN (*S*. *marcescens*), (**f**) DNP vs. ANP (*S*. *marcescens*), (**g**) DNP vs. DNN (*S*. *marcescens*), (**h**) DNP9 vs. NDNN9 (*S. marcescens*), (**i**) DNP vs. NDNN (*P. aeruginosa*).

**Figure 3:**
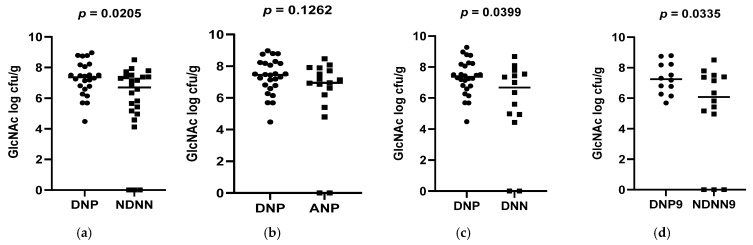
Abundance of N-acetylglucosamine in all selected bacteria (**a**) DNP vs. NDNN (**b**) DNP vs. ANP (**c**) DNP vs. DNN (**d**) DNP vs. NDNN at 9 months of age.

A total of 178 diarrheic infants were enrolled into the study and assayed for norovirus infection. Of the 178 infants, 26 were positive for norovirus and were, together with 26 non-diarrheic norovirus-negative from a cohort attending an immunization programme, 15 asymptomatic norovirus-positive and 15 diarrheic norovirus-negative infants, used to evaluate differences in levels of bacterial N-acetylglucosamine and selected LPS-rich bacteria. The results, expressed as a log colony-forming unit per gram (CFU/g), are shown in Figure 1, Figure 2 and Figure 3. The average levels of selected LPS gene targets were significantly high in DNP infants (6.17 ± 2.14 CFU/g) compared to their NDNN counterparts (4.13 ± 2.25 CFU/g), *p* = 0.003 (Figure 1a). Similarly, DNP infants harboured significantly higher levels of N-acetylglucosamine (7.33 ± 1.11 CFU/g) than NDNN infants (5.484 ± 2.18), *p* = 0.0205 (Figure 3a). Individually, DNP infants possessed significantly high levels of *S. marcescens* (8.47 ± 4.04 CFU/g) compared to their non-diarrheic norovirus-negative peers (5.52 ± 4.24 CFU/g), *p* = 0.0074 (Figure 2e). So too were the levels of *K. pneumoniae* in DNP infants (5.39 ± 2.28 CFU/g) versus NDNN infants (3.28 ± 1.86 CFU/g), *p* = 0.0264 (Figure 2a). There was no significant difference in levels of *P. aeruginosa* between the two study groups (*p* = 0.1629) (Figure 2i).

When compared to infants with diarrhea due other enteric pathogens, the mean abundance of all selected LPS gene targets were significantly high in DNP infants (6.17 ± 2.14 CFU/g) compared to DNN infants (3.67 ± 2.05 CFU/g), *p* = 0.0023 (Figure 1c). Similarly, DNP infants contained significantly higher levels of N-acetylglucosamine (7.33 ± 1.11 CFU/g) than DNN infants (5.72 ± 2.44 CFU/g), *p* = 0.0399 (Figure 3c). However, there was no difference in the mean abundance of all selected LPS gene targets as well as N-acetylglucosamine in DNP and their asymptomatic (ANP) counterparts, *p* = 0.3480 and 0.1262, respectively (Figure 1b and Figure 2b). Individually, significantly higher levels of *S*. *marcescens* and *K pneumoniae* were observed in DNP infants (8.47 ± 4.04 CFU/g and 5.39 ± 2.28 CFU/g, respectively) compared to their DNN counterparts (4.19 ± 2.75 CFU/g and 3.16 ± 2.09 CFU/g), *p* = 0.0007 and 0.0342, respectively (Figure 2g,c). Except for *S*. *marcescens* (*p* = 0.0330) (Figure 2f), there were no significant differences between the levels of N-acetylglucosamine and *K pneumoniae* in DNP and ANP infants, *p* = 0.1262 and 0.9058, respectively (Figure 1b and Figure 2b).

When abundance was stratified according to age, 3.5 and 9 month-old DNP infants contained significantly higher levels of all selected LPS-rich bacteria (5.92 ± 2.56 CFU/g) and (6.69 ± 2.11 CFU/g), respectively, compared to their NDNN counterparts (3.73 ± 2.76 CFU/g) and (4.16 ± 3.02 CFU/g), *p* = 0.0331 and 0.0210, respectively (Figure 1d,e). The levels of N-acetylglucosamine were only significantly higher in 9-month-old DNP (6.69 ± 2.34 CFU/g) than 9-month-old NDNN infants (6.02 ± 2.13 CFU/g), *p* = 0.0335 (Figure 3d). So too was the level of *S. marcescens* in 9-month-old DNP infants (8.36 ± 3.42 CFU/g) compared to non-diarrheic norovirus-negative peers and (4.71 ± 3.08 CFU/g), *p* = 0.0140 (Figure 2h). On the other hand, 3.5-months-old diarrheic norovirus-positive infants harboured significantly higher levels of *K. pneumoniae* (4.34 ± 2.65 CFU/g) than non-diarrheic norovirus-negative infants (2.08 ± 1.57 CFU/g), *p* = 0.0169 (Figure 2d).

### 3.3. Expression of the Select LPS, N-Acetylglucosamine and TLR4 in Stool mRNA Samples

**Figure 4:**
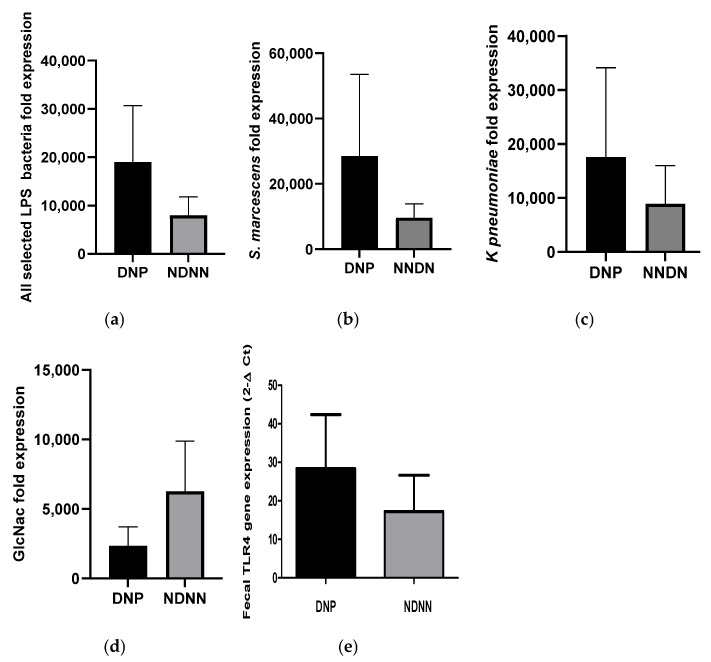
Expression of LPS genes of selected LPS-rich bacteria, N-acetylglucosamine and TLR4 between DNP and NDNN study groups: (**a**) mean of all selected LPS genes, (**b**) *S. marcescens*, (**c**) *K. pneumoniae*, (**d**) N-acetylglucosamine, and (**e**) TLR4.

The expression levels of the bacterial LPS genes (*Waa*E for *K. pneumoniae*, and *kdtX* for *S. marcescens* as well as *Glm*U for bacterial N-acetylglucosamine) were compared between DNP and NDNN samples. There was a 7-fold increase in average expression of all selected LPS genes in DNP infants compared to their NDNN counterparts (Figure 4a). Individually, there was a 4.7- and 17-fold increase in average expression of *Waa*E and *Kdt*X genes, respectively, in DNP infants compared to NDNN infants (Figure 4b,c). On the other hand, the average expression of GlmU decreased 10-fold in DNP compared to their NDNN counterparts (Figure 4d). The expression of TLR4 in fecal samples was consistent with the abundance of the LPS as it increased 2.5-fold in DRP children compared to NDRN children (Figure 4e).

### 3.4. Association Between the Abundance of Bacterial LPS and Norovirus Diarrhea

The average levels of selected LPS-rich bacteria in stool samples of DNP and NDNN were used as a continuous variable to evaluate its association with norovirus diarrhea. Infants harbouring increased levels of bacterial LPS were more likely to have norovirus diarrhea compared to those with low levels of the compound (odds ratio [OR] = 1.51, 95% confidence interval [CI] = 1.14–2.01, *p* = 0.033). Other potential confounding factors such as sex, mode of delivery, and feeding type did not contribute in predicting the levels of selected LPS gene target in the infants (OR = 0.899, 95% CI: 0.6832–1.1829, *p* = 0.447; OR = 1.037, 95% CI: 0.7638–1.4092, *p* = 0.814; and OR = 0.8726, 95% CI: 0.4966–1.5333, *p* = 0.637, respectively.

## 4. Discussion

The study found significantly higher levels of bacterial N-acetylglucosamine and selected LPS-rich bacteria in DNP children compared to their NDNN counterparts. Lack of homology between LPS genes in different bacteria rendered designing of primers to target all bacterial LPS in stool samples impossible; as such the study used the three selected bacteria as representative of LPS-rich bacteria. Nevertheless, the findings suggest that elevated levels of bacterial LPS may play a role in norovirus infection and diarrhea. In fact, logistical regression analysis of bacterial counts and norovirus diarrhea indicated an association between the level of fecal bacterial LPS and the disease. Elevated levels of LPS-rich bacteria mean more noroviruses will be able to bind to LPS, survive harsh intestinal conditions and become facilitated to attach and infect the target cells (affording the virus more time to reach its target) [4,5] and cause diarrhea. The mechanism through which bacterial LPS stabilizes norovirus is not fully explained but it is thought the negative charge of the LPS (due to phosphate group) interacts with the charged region of norovirus capsid, thereby preventing viral conformational change essential for viral inactivation and providing viral stability. Nevertheless, our findings are the first to show association between abundance of LPS and norovirus infection. However, a recent similar study observed that the accumulation of LPS secreted by enriched Gram-negative bacteria was a risk factor for rotavirus colonization in young adults [15].

The abundance of LPS-rich bacteria was assayed using qPCR, which gives an estimate of genome copy numbers but not viable counts of the bacteria. To estimate the level of viable bacteria, the study assayed the expression of gene coding for LPS genes of the selected bacteria between DNP and NDNN infants. Consistent with the abundance of the bacterial LPS gene targets, the average expression of all selected LPS genes were 7-fold higher in DNP than in NDNN. In contrast, the expression of the gene coding for N-acetylglucosamine was much less in DNP infants compared to NDNN infants, suggesting that N-acetylglucosamine from non-viable cells constituted much of total bacterial N-acetylglucosamine assayed. Nonetheless, studies have shown that enhancement of enteric viral infectivity does not require viable bacteria as lipopolysaccharides alone have been shown to directly bind to enteric viruses and promote infection [4].

The mean levels of selected LPS gene targets were high regardless of whether the infection was symptomatic or asymptomatic. It is not known whether the asymptomatic norovirus-positive infants were truly asymptomatic as human studies have indicated [16] or were merely recovering from norovirus diarrhea at the time of sampling that may indicate the continuing fecal norovirus shedding from past infection. Nevertheless, the observation suggests that elevated levels of bacterial LPS could be a susceptibility factor for both norovirus diarrhea and asymptomatic norovirus infection.

The expression of TLR4 gene in the current study corresponded with abundance or expression of selected bacterial LPS. This is consistent with studies in mice, which have shown that LPS activation of the TLR4 gene proceeds in a dose-dependent manner [17]. Immune signalling of LPS occurs via TLR4 and its increase in expression in stool samples of DNP children observed in the current study suggest that LPS may have a role in norovirus infection. The induction of TLR4 can lead to the release of proinflammatory cytokines such as interleukin 6 and 8 (IL-6 and 8) that have been associated with norovirus infections [18]. It is possible that an increase in expression of TLR4 in DRP as the result of higher abundance of LPS resulted in increased levels of IL-6 and IL-8 that created a conducive environment for norovirus infection and diarrhea.

Composition of the gut bacteria can temporarily change due to several physiological conditions including diarrhea. Diarrhea destabilizes the gut environment by increasing bowel movement and fluid secretion that can lead to gut microbiome dysbiosis [19]. The significant differences in abundance of selected bacteria LPS gene targets between DNP and DNN infants observed in the current study suggest that diarrhea could not be the cause of elevated levels of the selected LPS-rich bacteria in DNP infants since both study groups had diarrhea.

When age was stratified, the study observed that differences in levels of fecal bacterial N-acetylglucosamine and LPS-rich *S. marcescens* in diarrheic norovirus-positive and non-diarrheic norovirus-negative infants were notable in 9-month-old infants compared to their 3.5-month old counterparts. The levels of *K. pneumoniae*, on the other hand, were more significant in 3.5-month-old infants than 9-month-olds. During the first two years of life, the composition of gut bacteria is dynamic [19], and the observation in the current study suggests that at any particular time, elevated levels of these LPS-rich bacteria or bacterial N-acetylglucosamine could predispose infants to norovirus diarrhea.

The study also observed that sex, mode of delivery, and feeding type did not contribute significantly in predicting the levels of bacterial N-acetylglucosamine and selected LPS-rich bacteria among the study infants. There have been conflicting reports about differences in gut bacterial abundance between males and females, with some suggesting there is a difference between the two [20,21,22,23], while others suggest none [24]. The observation in the current study is in agreement with the latter and could be one of the reasons why both genders are equally affected by norovirus [22]. As the levels and diversity of intestinal bacteria during the first year of life is dynamic and unsteady [19], it is not surprising that the abundance of bacterial N-acetylglucosamine and the selected LPS-rich bacteria in infants’ fecal samples could be not predicted according the three confounding factors.

The study had limitations, the first being the small number of samples used to evaluate the differences in levels of bacterial N-acetylglucosamine and selected LPS-rich bacteria between the two study groups. Further studies, with a large sample size, are warranted to validate the current study observations. Secondly, the study population comprised only black South African infants, and since there are reported differences in diversity and composition of bacterial species and strains among different ethnic populations, it would be intriguing to find out the levels of bacterial N-acetylglucosamine and selected LPS-rich bacteria in other ethnic or racial groups. Thirdly, due to little homology of gene coding for the synthesis of LPS, quantification of LPS was only performed for selected LPS-rich bacteria and future studies involving quantification of overall bacterial LPS, possibly through chromatography, is required. Lastly, stool samples are not true indicators of the abundance of bacteria in the intestines where norovirus are suspected to primarily infect. However, they are frequently used as proxies for gut bacteria as they can be collected naturally and non-invasively.

In summary, the study has shown that diarrheic norovirus-positive infants contained significantly high levels of bacterial N-acetylglucosamine and selected LPS-rich bacteria compared to their non-diarrheic norovirus-negative counters. In addition, the expression of LPS signalling molecules increased 2.5-fold in infants with norovirus diarrhea compared to the controls. Infants possessing elevated levels of selected LPS-rich bacteria were more likely to have norovirus diarrhea. The results suggest the possible role of the abundance of bacterial LPS in norovirus infection.

## Figures and Tables

**Table 1:** Primers and probes used in this study.

Species/Compound	Name and Sequences	Gene	Ref
*P. aeruginosa*	WaaLF-CCAGATCAGCGAGCATCCAT WaaLR-CGAAAAGCACACCCAGTTCG WaaLP-Texas red-CGGCTACGATCATCCGAT- BHQ-2	*Waa*L	This study
*S. marcescens*	WaaF-TCGACGGTAAACAGGGGTTG WaaR-TCACTTCTCTGAGTAGTTGCGG WaaP-Hex-TAGCGGTGGTCAACGCGCAATATA-BHQ-1	*kdt*X	This study
*K. pneumoniae*	WaaEF-TCGTTATAGCGGTAACGGGC WaaER-TCGCCCGCCGTAACTATTTT WaaEP-Hex-ATACCAACCGCTGTGGCGCATAAA-BHQ-1	*Waa*E	This study
N-acetylglucosamine	GlmU-F-GTGATGTAGTATTCGCCCTGAG GlmU-R-AAGATGCCACCGACGAGCAG GlmU-P FAM-TTGTTGGTCAGCTTCGCCAG-BHQ-1	*Glm*U	This study
Toll-like receptor 4	TLR4 F-GATTGCTCAGACCTGGCAGTT TLR4 R-GTCCTCCCACTCCAGGTAAGT	TLR4	This study

**Table 2:** Demographics and other baseline characteristics between diarrheic norovirus-positive (DNP) and non-diarrheic norovirus-negative (NNDN) infants.

Characteristics		DNP n (%)	NDNN n (%)	*p* Value
Gender	Female Male	12 (46) 14 (54)	10 (39) 16 (61)	0.5745
Age	3.5 months 9 months	14 (54) 12 (46)	11 (42) 15 (58)	0.4050
Mode of delivery	Natural birth Caesarean	20 (77) 6 (23)	17 (65) 9 (35)	0.3584
Feeding type	Breast milk Formula	25 (96) 1 (04)	23 (88) 3 (12)	0.2979

## Data Availability

The raw data supporting the conclusions of this article will be made available by the corresponding author on request.
